# Population effectiveness of endoscopy screening for mortality reduction in gastric cancer

**DOI:** 10.1002/deo2.296

**Published:** 2023-09-19

**Authors:** Naoki Ishii, Yasutoshi Shiratori, Masahiro Ishikane, Fumio Omata

**Affiliations:** ^1^ Division of Gastroenterology Tokyo Shinagawa Hospital Tokyo Japan; ^2^ Division of Gastroenterology Maimonides Medical Center New York USA; ^3^ Disease Control and Prevention Center National Center for Global Health and Medicine Tokyo Japan; ^4^ Yokohama Konandai Clinic Kanagawa Japan

**Keywords:** early detection of cancer, early diagnosis, endoscopy, gastric cancer, stomach neoplasms

## Abstract

**Objectives:**

No randomized controlled trials have compared endoscopic screening with no screening for gastric cancer on an intention‐to‐screen basis, and the population‐based evidence is insufficient. This study aimed to identify factors contributing to the population effectiveness of cancer screening, estimate the number needed to screen (NNS) to reduce one gastric cancer‐related death, and evaluate the expected mortality‐rate reduction in endoscopic screening for gastric cancer in 184 countries.

**Methods:**

Factors contributing to the attributable risk, NNS, and mortality‐rate reduction were identified. A rapid review was performed in PubMed to estimate the pooled relative risk of endoscopic screening compared to that of no screening for mortality reduction. NNSs and mortality‐rate reduction were estimated using the pooled relative risk and GLOBOCAN data.

**Results:**

The crude mortality rate, the effectiveness of the screening modality, and the screened rate contributed to the attributable risk, NNS, and mortality‐rate reduction in cancer screening. The pooled relative risk was 0.58 in endoscopy screening compared to that in no screening. NNSs and expected mortality‐rate reduction differed across countries and ranged from 2522 to 91,575 and 0.2 to 7.9 (per 100,000 individuals) for the screened rate of 20%, respectively.

**Conclusions:**

In addition to the effectiveness of the used modality, the disease burden and screened rate were important in the population effectiveness of cancer screening. Regarding the high NNSs and the low expected mortality‐rate reduction, population‐based endoscopic screening seems not to be effective in many countries, and these results are meaningful in decision‐making regarding the introduction of endoscopic screening.

## INTRODUCTION

**FIGURE 1:**
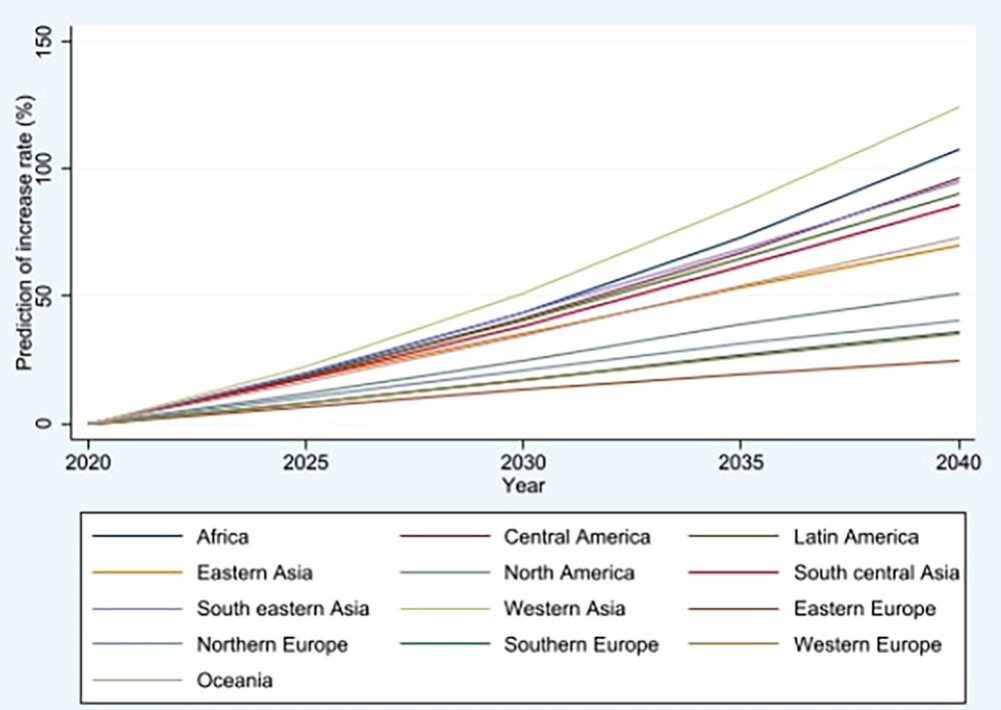
Prediction of increased rate for the number of gastric cancer‐related deaths from 2020 to 2040 in 12 regions. Note. This figure was created based on the GLOBOCAN data.^2^ Cancer Tomorrow. Estimated number of deaths from 2020 to 2040, stomach, both sexes, age 50–85+. URL: https://gco.iarc.fr/tomorrow/en/dataviz/isotype?cancers=7&single_unit=50000&types=1&age_start=10 [accessed April 23, 2023].

Gastric cancer is the fourth leading cause of cancer‐related deaths globally.^1^ According to the GLOBOCAN data produced by the International Agency for Research on Cancer, the number of gastric cancer‐related deaths is expected to increase from 2020 to 2040 in all regions (Table S1 and Figure 1).^1^, ^2^ The prognosis of gastric cancer is favorable if diagnosed and treated at an early stage^3^, ^4^; thus, population‐based cancer screening has been introduced in high‐burden countries such as Japan and the Republic of Korea, in which endoscopic screening was also recommended as one of the screening modalities.^5^, ^6^ Recently, the effectiveness of endoscopic screening in reducing gastric cancer mortality has been reported^6^, ^7^; however, the evidence is based on cohort studies and case‐control studies, and no randomized controlled trials (RCTs) have compared endoscopic screening with no screening for gastric cancer on an intention‐to‐screen basis. Even in the updated version of the Japanese Guidelines for Gastric Cancer Screening,^5^ the effectiveness of endoscopy screening was referred from a few observational studies, and a meta‐analysis was not used for the evaluation of endoscopy effectiveness for mortality reduction. Therefore, the attributable risk between endoscopic screening and no screening for cancer mortality and the number needed to screen (NNS) required to reduce one gastric cancer‐related death remain insufficient. Nevertheless, NNS and the expected mortality‐rate reduction by endoscopy screening are required for the introduction of endoscopic screening, especially in countries with a high gastric cancer burden.

This study aimed to identify the factors contributing to population‐based cancer screening and to estimate the NNS and expected mortality‐rate reduction during endoscopic screening for gastric cancer in 184 countries using GLOBOCAN data.

## METHODS

### Identification of the factors contributing to population‐based cancer screening

The factors contributing to the population‐based cancer screening were identified from the attributable risk, the NNS, and the mortality rate.

The NNS was divided by the attributable risk. Therefore, the factors contributing to the NNS were identified from the attributable risk (Table 1).

**TABLE 1:** Identification of the factors contributing to population‐based cancer screening

Viewpoint from attributable risk (AR) and number needed to screen (NNS)
Attributable risk (AR) and number needed to screen (NNS) were calculated as follows: $\begin{array}{c} AR\;=\text{mortality rate in the unscreened population}(MU)-\text{mortality rate in the screened population}(MS)\\ \;\;=MU-MU\times \text{relative risk}(RR)\\ \;\;=MU\times (1-RR)(\text{/100,000 persons}) \end{array}$ $\begin{array}{c} NNS=1/AR(\text{/100,000 persons})\\ \;\;\;=1/(MU\times (1-RR))(\text{/100,000 persons})\\ \;\;\;=100,000/(MU\times (1-RR))\\ \;\;\;=100,000/(\text{crude mortality rate}(CMR)\times (1-RR)) \end{array}$

Viewpoint from mortality rate in the total population
The mortality rate in the total population (MT) (cases/100,000 persons) was calculated as follows: $\begin{array}{c} \text{The mortality rate in the total population}\;(\mathrm{MT})\;(\text{cases/100,000 persons})\\ \;\;\;=(\text{mortality rate in the unscreened population}\;(\mathrm{MU})\times \text{unscreened population}\;(\text{persons})\\ \;\;\;+\;\text{mortality rate in the screened population}\;(\mathrm{MS})\times \text{screened population}\;(\text{persons}))\;\text{/ 100,000}\\ \;\;\;=(\mathrm{MU}\times \mathrm{TP}\times (1-\text{screened rate}\;(\mathrm{SR}))+\mathrm{MU}\times \mathrm{RR}\times \mathrm{TP}\times \mathrm{SR})\;\text{/ 100,000}\\ \;\;\;=\mathrm{MU}\times \mathrm{TP}\times ((1-\mathrm{SR})+\mathrm{RR}\times \mathrm{SR})\text{/100,000}\\ \;\;\;=\mathrm{MU}\times \mathrm{TP}\times (1-\mathrm{SR}\times (1-\mathrm{RR}))\text{/100,000}\\ \;\;\;=(\mathrm{MU}\times \mathrm{TP}/100,000)\times (1-\mathrm{SR}\times (1-\mathrm{RR}))\\ \;\;\;=\text{mortality rate in the countries in which endoscopy screening was not adopted}\times (1-\mathrm{SR}\times (1-\mathrm{RR}))\\ \;\;\;=\mathrm{CMR}\times (1-\mathrm{SR}\times (1-\mathrm{RR})) \end{array}$ Mortality‐rate reduction = CMR × SR× (1 ‐ RR))

*Notes*: Total population (TP) = unscreened population + screened population.

Unscreened population = TP × (1 ‐ screened rate [SR]).

Screened population = TP × SR.

Relative risk (RR) = mortality rate in screened population (MS)/mortality rate in unscreened population (MU).

Attributable risk (AR) = MU – MS.

Abbreviations: AR, attributable risk; CMR, crude mortality rate; MS, mortality rate in the screened population (cases/100,000 persons); MT, mortality rate in the total population (cases/100,000 persons); MU, mortality rate in the unscreened population (cases/100,000 persons); NNS, number needed to screen; RR, relative risk; SR, screened rate; TP, total population (persons).

Next, the factors contributing to mortality‐rate reduction were identified from the mortality rate in the total population (cases/100,000 persons; Table 1).

### Data extraction of crude mortality rates in 184 countries

Data on the number of gastric cancer‐related deaths, crude mortality rates (CMR), and age‐standardized mortality rates in 2020 in 184 countries were extracted from GLOBOCAN data (Table S2).^8^ Endoscopic screening is performed as a population‐based gastric cancer screening program for people aged 50 years in Japan^5^; the mortality rate of patients aged 50 years was used in the present analysis.

### Estimation of the pooled relative risk in endoscopy screening

Naoki Ishii and Yasutoshi Shiratori independently performed a rapid review of the effectiveness of endoscopic screening compared to that of no screening for mortality reduction in gastric cancer in PubMed to estimate the pooled relative risk (RR). If no agreement could be reached after the rapid reviews, it was planned that a third author (Masahiro Ishikane or Fumio Omata) would decide to obtain the consensus. Because there were no RCTs, systematic reviews of observational studies were searched on April 23, 2023, using the following specific search terms: ((“Stomach Neoplasms”[MeSH Terms] OR ((“gastric”[Title/Abstract] OR “stomach”[Title/Abstract]) AND (“cancer*”[Title/Abstract] OR “neoplasm*”[Title/Abstract] OR “tumor*”[Title/Abstract] OR “tumour*”[Title/Abstract]))) AND (“endoscopie”[All Fields] OR “endoscopy”[MeSH Terms] OR “endoscopy”[All Fields] OR “endoscopies”[All Fields] OR “endoscopy s”[All Fields]) AND (“diagnosis”[MeSH Subheading] OR “diagnosis”[All Fields] OR “screening”[All Fields] OR “mass screening”[MeSH Terms] OR (“mass”[All Fields] AND “screening”[All Fields]) OR “mass screening”[All Fields] OR “early detection of cancer”[MeSH Terms] OR (“early”[All Fields] AND “detection”[All Fields] AND “cancer”[All Fields]) OR “early detection of cancer”[All Fields] OR “screen”[All Fields] OR “screenings”[All Fields] OR “screened”[All Fields] OR “screens”[All Fields] OR (“mortality”[MeSH Terms] OR “death rate”[Text Word]))) AND ((systematicreview[Filter]) AND (2000/1/1:2023/4/23[pdat])).

The pooled RR comparing endoscopic screening with no screening for gastric cancer mortality in a systematic review and meta‐analysis was used for the estimation of the NNS and expected mortality‐rate reduction in 184 countries.

### Calculation of the NNS and expected mortality‐rate reduction

The NNS and expected mortality‐rate reduction were calculated using the mortality rates from the GLOBOCAN data and the pooled RR. A screened rate (SR) of 20% by endoscopy was used as an example in the present study based on the previous reports (18.6‐28.2%).^7^, ^9^ In addition to endoscopy, fluoroscopy has been used as a screening modality for gastric cancer in Japan,^5^ and the SR of gastric cancer screening by fluoroscopy or endoscopy aged 50 years or older within 2 years was 42.4% in Japan in 2022.^10^

### Data analysis

Data analyses were performed using STATA version 16 software (StataCorp). This study was performed based on GLOBOCAN data, and the requirement for informed consent and approval from the institutional review board was waived.

## RESULTS

### Factors contributing to the NNS

The formulae for calculating the NNS are shown in Table 1.

$$\begin{array}{ccc} NNS & = & \text{100,000 divided by}\\ & & \left(\left(\text{mortality rate in the unscreened population}\right)\right.\\ & & \left.\times \left(1-RR\right)\right). \end{array}$$

Endoscopic screening has not been adopted in most countries other than Japan and the Republic of Korea. The mortality rates in the unscreened population could be considered as the CMRs of each country obtained from the GLOBOCAN data.

$$NNS=\text{100,000 divided by}\left(CMR\times \left(1-RR\right)\right).$$

An increase in the CMR or the disease burden and a decrease in the RR could lead to a reduction in the NNS.

### Factors contributing to the mortality‐rate reduction

The formulae for calculating the expected mortality‐rate reduction are shown in Table 1.

$$\begin{array}{ccc} & & \text{The mortality rate in the total population}\\ & & \left(cases/\text{100,000 persons}\right)=CMR\times \left(1-SR\times \left(1-RR\right)\right). \end{array}$$

$$\begin{array}{ccc} & & \text{Therefore,the expected mortality}-\text{rate reduction}\\ & & =CMR\times SR\times \left(1-RR\right). \end{array}$$

(1 ‐ RR) can be considered the effectiveness of the screening modality at the individual level. In addition to the effectiveness of the screening modality, the mortality rate was reduced by the increase in the SR.

### Pooled RR in endoscopic screening for gastric cancer‐related mortality

In total, 127 papers were searched, two of which were systematic reviews of endoscopic screening for gastric cancer mortality reduction.^11^, ^12^ There were two fully reviewed studies comparing endoscopic screening with no screening for mortality reduction, and a meta‐analysis was not performed in the study by Faria et al.^12^ In addition to the two studies included by Faria et al.^12^, Zhang et al. included a total of six cohort studies and four nested case‐control studies and performed a meta‐analysis. Therefore, the pooled RR of 0.58 in the study by Zhang et al.^11^ was adopted in the present study.

### Estimated NNSs and expected mortality‐rate reductions in 184 countries

CMRs and the pooled RR of 0.58 were used for calculating the NNS (Table 1). The CMRs, the pooled RR of 0.58, and the SR of 20% were used for the estimation of the mortality‐rate reduction (Table 1).

In Mongolia, the CMR was 94.4 (cases/100,000 persons).
The NNS in Mongolia was calculated by 100,000 divided by (94.4 × (1 – 0.58)).
The expected mortality‐rate reduction by an SR of 20% in Mongolia was calculated by 94.4 × 0.2 × (1 – 0.58) (cases/100,000 persons).

The estimated NNSs and the expected mortality‐rate reduction by an SR of 20% in 184 countries are shown in Table 2.

**TABLE 2:** The estimated number needed to screen (NNS) and the expected mortality‐rate reduction by the screened rate of 20% in 184 countries.

Countries	CMR	NNS	Mortality‐rate reduction by SR of 20%	Countries	CMR	NNS	Mortality‐rate reduction by SR of 20%	Countries	CMR	NNS	Mortality‐rate reduction by SR of 20%	Countries	CMR	NNS	Mortality‐rate reduction by SR of 20%

Mongolia	94.4	2522	7.9	North Macedonia	36.9	6452	3.1	Israel	21.6	11,023	1.8	Bahrain	13.9	17,129	1.2
Japan	75.7	3145	6.4	Poland	36.5	6523	3.1	Nepal	21.5	11,074	1.8	Sweden	13.7	17,379	1.2
China	73.9	3222	6.2	Panama	36.1	6595	3.0	Mexico	21.1	11,284	1.8	Cameroon	13.7	17,379	1.2
Tajikistan	73.8	3226	6.2	Albania	36	6614	3.0	Jordan	20.9	11,392	1.8	Egypt	13.4	17,768	1.1
Bhutan	70.2	3392	5.9	Slovenia	35.1	6783	2.9	Morocco	20.8	11,447	1.7	Cambodia	13.3	17,902	1.1
The Islamic Republic of Iran	69.9	3406	5.9	Republic of Korea	34.6	6881	2.9	Cuba	20.6	11,558	1.7	Angola	13.2	18,038	1.1
Cabo Verde	66.7	3570	5.6	Hungary	34.3	6942	2.9	Mauritania	20.4	11,671	1.7	Canada	12.9	18,457	1.1
Kyrgyzstan	62.3	3822	5.2	Bulgaria	34.3	6942	2.9	Ireland	20.2	11,787	1.7	Iceland	12.9	18,457	1.1
Peru	60.7	3922	5.1	Slovakia	33	7215	2.8	Burundi	20.2	11,787	1.7	Puerto Rico	12.8	18,601	1.1
Chile	55.7	4275	4.7	Uzbekistan	32.7	7281	2.7	Algeria	19.7	12,086	1.7	Malawi	12.7	18,748	1.1
Vietnam	54.6	4361	4.6	La Reunion, France	32.2	7394	2.7	Barbados	19.7	12,086	1.7	Uganda	12.6	18,896	1.1
Belarus	53.1	4484	4.5	Honduras	31.8	7487	2.7	Bahamas	19.5	12,210	1.6	South Africa	12.5	19,048	1.1
Haiti	53.1	4484	4.5	Brunei Darussalam	31.6	7535	2.7	Guinea‐Bissau	18.7	12,732	1.6	Libya	12.5	19,048	1.1
Estonia	52.8	4509	4.4	The Plurinational State of Bolivia	31.3	7607	2.6	Bangladesh	18.6	12,801	1.6	Zambia	12.4	19,201	1.0
Costa Rica	52.5	4535	4.4	Italy	31.1	7656	2.6	Republic of Congo	18.2	13,082	1.5	Burkina Faso	12.2	19,516	1.0
Armenia	52.4	4544	4.4	Serbia	30.8	7730	2.6	France	18	13,228	1.5	Niger	12.1	19,677	1.0
The Democratic Republic of Korea	52.2	4561	4.4	New Caledonia, France	30.5	7806	2.6	Democratic Republic of Congo	17.4	13,684	1.5	Thailand	11.9	20,008	1.0
Latvia	51.5	4623	4.3	Rwanda	29.5	8071	2.5	Finland	17.3	13,763	1.5	Ethiopia	11.9	20,008	1.0
Ecuador	51	4669	4.3	Uruguay	29.4	8098	2.5	Denmark	17.3	13,763	1.5	Australia	11.8	20,178	1.0
Martinique, France	51	4669	4.3	Greece	28.8	8267	2.4	Togo	17.3	13,763	1.5	Fiji	11.8	20,178	1.0
Russian Federation	50.9	4678	4.3	Singapore	28.7	8296	2.4	Gaza Strip and West Bank	17.3	13,763	1.5	Eritrea	11.5	20,704	1.0
Lithuania	50.6	4705	4.3	Oman	28.5	8354	2.4	Cote d'ivoire	17.2	13,843	1.4	Chad	11.2	21,259	0.9
Kazakhstan	50.4	4724	4.2	Papua New Guinea	28.3	8413	2.4	South Sudan	17.1	13,924	1.4	Lebanon	11	21,645	0.9
Guatemala	50.2	4743	4.2	Cyprus	27.9	8534	2.3	Suriname	17.1	13,924	1.4	The Republic of the Gambia	10.9	21,844	0.9

Myanmar	49.5	4810	4.2	Paraguay	27.7	8595	2.3	Switzerland	17	14,006	1.4	Djibouti	10.6	22,462	0.9
Portugal	49.4	4820	4.1	Malta	27.6	8627	2.3	The Netherlands	16.9	14,088	1.4	Qatar	10.4	22,894	0.9
Turkey	48.8	4879	4.1	Montenegro	27.4	8690	2.3	Somalia	16.9	14,088	1.4	Equatorial Guinea	10.4	22,894	0.9
Afghanistan	47	5066	3.9	Benin	27.2	8754	2.3	Madagascar	16.8	14,172	1.4	Tunisia	9.9	24,050	0.8
Mali	46.8	5088	3.9	French Polynesia	27.1	8786	2.3	Liberia	16.8	14,172	1.4	Guyana	9.7	24,546	0.8
Colombia	46.4	5131	3.9	Dominican Republic	26.9	8851	2.3	Sierra Leone	16.6	14,343	1.4	Nigeria	9.4	25,329	0.8
Azerbaijan	46.3	5142	3.9	Yemen	26.3	9053	2.2	India	16.5	14,430	1.4	Sri Lanka	9.1	26,164	0.8
Guadeloupe, France	44.4	5363	3.7	French Guiana	26.3	9053	2.2	Belgium	16.5	14,430	1.4	United Arab Emirates	8.9	26,752	0.7
Bosnia and Herzegovina	42.9	5550	3.6	Spain	26.2	9088	2.2	United Kingdom	16.3	14,607	1.4	United States of America	8.8	27,056	0.7
Lao People's Democratic Republic	42.7	5576	3.6	Kenya	26.1	9122	2.2	Trinidad and Tobago	16.3	14,607	1.4	Sudan	8.3	28,686	0.7
Turkmenistan	41	5807	3.4	Brazil	26	9158	2.2	Iraq	15.9	14,975	1.3	Namibia	8.2	29,036	0.7
Romania	40.7	5850	3.4	Senegal	25.9	9193	2.2	United Republic of Tanzania	15.5	15,361	1.3	Gabon	7.9	30,139	0.7
Republic of Moldova	39.6	6013	3.3	Argentina	25.7	9264	2.2	Guinea	15.3	15,562	1.3	Saudi Arabia	7.7	30,921	0.6
Croatia	39.5	6028	3.3	The Bolivarian Republic of Venezuela	25.6	9301	2.2	Malaysia	15.1	15,768	1.3	Kuwait	6.8	35,014	0.6
Georgia	38.9	6121	3.3	Jamaica	25.1	9486	2.1	Pakistan	15	15,873	1.3	Eswatini	6.8	35,014	0.6
Ukraine	38.2	6233	3.2	Guam	24	9921	2.0	Ghana	15	15,873	1.3	Lesotho	6.6	36,075	0.6
Sao Tome and Principe	38	6266	3.2	Germany	23.6	10089	2.0	Timor‐Leste	14.8	16,088	1.2	Botswana	6.1	39,032	0.5
Nicaragua	37.5	6349	3.2	Mauritius	22.5	10582	1.9	Syrian Arab Republic	14.6	16,308	1.2	Comoros	5.2	45,788	0.4
Saint Lucia	37.5	6349	3.2	Belize	22.5	10582	1.9	Central African Republic	14.5	16,420	1.2	Solomon Islands	5	47,619	0.4
Zimbabwe	37.4	6366	3.1	Austria	21.8	10922	1.8	Norway	14.3	16,650	1.2	Indonesia	4.5	52,910	0.4
El Salvador	37.2	6400	3.1	Luxembourg	21.8	10922	1.8	New Zealand	14.3	16,650	1.2	Mozambique	2.7	88,183	0.2
Samoa	37	6435	3.1	Czechia	21.7	10972	1.8	Philippines	13.9	17,129	1.2	Vanuatu	2.6	91,575	0.2

*Note*: The mortality rate was the number of gastric cancer‐related deaths per 100,000 individuals.

Abbreviations: CMR, crude mortality rate; NNS, number needed to screen; SR, screened rate.

The NNSs and the mortality‐rate reduction differed across countries and ranged from 2522 to 91,575 and 0.2 to 7.9 (per 100,000 individuals), respectively. The expected mortality‐rate reduction would increase according to the SR.

## DISCUSSION

This study identified three important factors contributing to the population‐level effectiveness of cancer screening and is the first to estimate the NNS and mortality‐rate reduction in endoscopic screening for gastric cancer in 184 countries.

We proposed the following three important factors to be considered in population‐based cancer screening: (1) disease burden, CMR; (2) RR in screening modality; (3) SR. Previous studies have reported the effectiveness of endoscopy screening for the reduction of gastric cancer‐related mortality.^5^, ^6^, ^7^, ^9^, ^11^ However, the disease burden and the SR were not considered, and the evidence was limited to the individual level.^13^ The attributable risk between endoscopy‐screened and unscreened populations, rather than the RR or odds ratio (OR), is important as population‐level evidence,^13^ but there have been no RCTs elucidating these points. On the other hand, RCTs have been performed based on an intention‐to‐screen basis in colorectal cancer screening unlike in gastric cancer screening.^14^, ^15^, ^16^, ^17^ Population‐level evidence is required before endoscopic screening for mortality reduction as mass screening is introduced in countries, unlike opportunistic screening, and RCTs are required based on an intention‐to‐screen basis in gastric cancer screening.

In this study, we estimated the NNS calculated from the attributable risk and the expected mortality‐rate reduction in endoscopic screening for gastric cancer. The NNSs ranged from 2522 to 91,575 and varied across countries in the present study. Recently, Mizota et al. proposed 1,000 as the NNS threshold for the introduction of population‐based endoscopy screening.^18^ However, there were no countries in which the NNSs were less than 1,000 in the present study. The expected mortality‐rate reduction in the SR of 20% ranged from 0.2 to 7.9 (per 100,000 individuals) and differed across countries. We can estimate the expected mortality‐rate reduction for gastric cancer using the results in Table 2, multiplied by the anticipated SR in each country divided by 20%. The mortality‐rate reduction was influenced by the SR, in addition to the disease burden and screening modality effectiveness. The budget for cancer screening, the number of endoscopists and medical staff, and the size of endoscopy facilities can directly contribute to the SR and these factors should be considered for increasing the SR and the mortality‐rate reduction. Our results could contribute to decision‐making regarding the introduction of endoscopic screening in each country. Due to the high NNSs and the low expected mortality‐rate reduction, population‐based endoscopic screening seems not to be effective, and further studies of endoscopy screening may not be realistic in many countries.

The NNS and the estimated mortality‐rate reduction are expected to vary greatly between *Helicobacter pylori*‐positive and *H. pylori*‐negative populations, even in high‐burden countries.^19^, ^20^ Certainly, the effectiveness of mass screening has been decreasing in Japan in accordance with the decreasing trend in *H. pylori* infection.^21^ Recently, the ABC method, involving the combination of anti‐*H. pylori* immunoglobulin G (IgG) antibody and serum pepsinogen, which are targeted for *H. pylori* infection and *H. pylori*‐associated gastritis, respectively, have been used for risk stratification of gastric cancer development before endoscopic screening.^22^, ^23^ In addition, given that the SR is also important for mortality‐rate reduction at the population level (Table 1) and was not sufficient in Japan,^7^, ^9^, ^10^ the ABC method, which is less invasive, inexpensive, and convenient, could cover a larger population, stratify the high‐risk populations, increase the SR, and lead to further mortality reduction. Certainly, the sensitivity of anti‐*H. pylori* IgG for infection is <80%,^24^ and there might be a risk that *H. pylori*‐infected patients with less atrophy might be classified into the *H. pylori*‐negative group via the ABC method.^24^, ^25^ A simultaneous screening is a test in which two tests are administered simultaneously (Table 3).^26^ All subjects receive two tests. A simultaneous screening can increase net sensitivity.^26^ Because the urea breath test is sensitive to *H. pylori* infection,^24^ but more complex than the stool antigen test considering its use at the population level, the ABC method combined with the stool antigen test may be a suitable simultaneous test for the diagnosis of *H. pylori*‐associated gastritis.^25^ A sequential screening is a test in which two tests are administered sequentially (Table 3)^26^: All subjects receive test 1, less expensive and less invasive. The subjects with test 1‐positive receive test 2. A sequential screening can increase net specificity.^26^ Although we cannot evaluate and recommend more effective screening based on the data in this study, the combination of the *H. pylori*‐associated gastritis tests followed by endoscopy may be considered an effective sequential screening for gastric cancer. We should consider the cost‐effectiveness, feasibility of cancer screening, and unintended risks in low‐risk populations for the development of gastric cancer. Therefore, it is a future issue to conduct more effective population‐based screening.

**TABLE 3:** Multiple screening tests.

	Simultaneous screening	Sequential screening
Tests administered	Two tests were administered simultaneously. All subjects receive two tests.	Two tests were administered sequentially. All subjects receive test 1, less expensive and less invasive. The subjects with test 1‐positive receive test 2.
Net sensitivity	Increase	Decrease
Net specificity	Decrease	Increase

The present study has several limitations. First, we identified three factors only from the NNS, the attributable risk, and the mortality rate in the total population. Second, this study was based on GLOBOCAN data. Detailed data from individuals were not used for the analyses, and the ethnic composition of the population of each country, the locations, and the histological types of gastric cancer could not be considered for the assessment of the mortality rate for gastric cancer.^27^ Furthermore, all the included studies in the meta‐analysis for the estimation of the pooled RR were from Eastern Asia.^11^ There are many countries where the technology of endoscopy is not sufficiently widespread, and endoscopic skills and access to medical care are quite different across countries. Under these circumstances, it might be difficult to discuss risk reduction using the same formula for 184 countries. Third, the most important risk factors for gastric cancer development, *H. pylori* infection, and *H. pylori*‐associated gastritis,^19^, ^20^ were not considered.

In conclusion, the disease burden, screening modality effectiveness, and SR were important factors in the population‐level effectiveness of cancer screening. The NNS to reduce one gastric cancer‐related death and the expected mortality‐rate reduction were estimated in endoscopic screening for gastric cancer in 184 countries, which showed them to differ among countries. Regarding the high NNSs and the low expected mortality‐rate reduction, population‐based endoscopic screening seems not to be effective in many countries, and these results are meaningful in decision‐making regarding the introduction of endoscopic screening.

## CONFLICT OF INTEREST STATEMENT

None.

## Supporting information

Table S1. Estimated number of gastric cancer deaths aged 50 or over from 2020 to 2040 in 13 regions.
