# Supplementary material for: Population effectiveness of endoscopy screening for mortality reduction in gastric cancer
Source: DEN Open. 2023 Sep 19;4(1):e296. doi: 10.1002/deo2.296 (PMC10508325; doi:10.1002/deo2.296)
Supplement: Supplementary file 1 — Table S1. Estimated number of gastric cancer deaths aged 50 or over from 2020 to 2040 in 13 regions. [file DEO2-4-e296-s001.docx]

**Supplementary Table 1. Estimated number of gastric cancer deaths aged 50 or over from 2020 to 2040 in 13 regions.**

| **Year** | **Eastern Asia** | **South central Asia** | **Latin America** | **Eastern Europe** | **South eastern Asia** | **Africa** | **Southern Europe** | **Western Europe** | **Western Asia** | **North America** | **Central America** | **Northern Europe** | **Oceania** |
| --- | --- | --- | --- | --- | --- | --- | --- | --- | --- | --- | --- | --- | --- |
| 2020 | 410657 | 103888 | 47618 | 46998 | 28575 | 22581 | 20616 | 16973 | 15509 | 12264 | 9358 | 7428 | 1689 |
| 2025 | 483586 | 122703 | 56512 | 50069 | 34408 | 26940 | 22321 | 18362 | 19009 | 13725 | 11116 | 8183 | 1964 |
| 2030 | 556848 | 143890 | 66757 | 53087 | 40927 | 32364 | 24197 | 19824 | 23456 | 15325 | 13201 | 8993 | 2275 |
| 2035 | 629907 | 167823 | 78328 | 55918 | 48193 | 39055 | 26191 | 21445 | 28784 | 17022 | 15656 | 9769 | 2605 |
| 2040 | 699620 | 193557 | 90779 | 58453 | 55663 | 46976 | 28093 | 22987 | 34804 | 18515 | 18380 | 10464 | 2927 |

**Supplementary Table 2. Estimated number of gastric cancer deaths and crude and age-standardized mortality rates in 184 countries in 2020**

| **Country** | **Number** | **CMR** | **ASR** | **Country** | **Number** | **CMR** | **ASR** | **Country** | **Number** | **CMR** | **ASR** | **Country** | **Number** | **CMR** | **ASR** |
| --- | --- | --- | --- | --- | --- | --- | --- | --- | --- | --- | --- | --- | --- | --- | --- |
| China | 351063 | 73.9 | 73.1 | Nepal | 1039 | 21.5 | 21.3 | Cote d’ivoire | 442 | 17.2 | 18.8 | Congo, Republic of | 105 | 18.2 | 21.6 |
| India | 44273 | 16.5 | 16.8 | Hungary | 1299 | 34.3 | 26.1 | Armenia | 477 | 52.4 | 46.2 | Cyprus | 108 | 27.9 | 22.4 |
| Japan | 45393 | 75.7 | 38.8 | Guatemala | 1180 | 50.2 | 44.5 | Syrian Arab Republic | 381 | 14.6 | 14.9 | Bhutan | 90 | 70.2 | 69.3 |
| Russian Federation | 26256 | 50.9 | 44.1 | Greece | 1296 | 28.8 | 18.8 | Uganda | 419 | 12.6 | 14 | Mozambique | 75 | 2.7 | 2.9 |
| Brazil | 14126 | 26 | 24.2 | The Netherlands | 1200 | 16.9 | 12.3 | Nicaragua | 406 | 37.5 | 36.3 | France, La Reunion | 93 | 32.2 | 27.6 |
| Viet Nam | 12622 | 54.6 | 54.1 | Azerbaijan | 1139 | 46.3 | 49.3 | Turkmenistan | 420 | 41 | 44 | Mauritius | 92 | 22.5 | 20.9 |
| Iran, Islamic Republic of | 11844 | 69.9 | 71.8 | Malaysia | 1013 | 15.1 | 15 | Cambodia | 353 | 13.3 | 14.3 | France, Martinique | 88 | 51 | 32.7 |
| United States of America | 10384 | 8.8 | 7.1 | Tajikistan | 923 | 73.8 | 88.7 | Finland | 403 | 17.3 | 12.1 | Central African Republic | 65 | 14.5 | 15.8 |
| Turkey | 9728 | 48.8 | 45.4 | Australia | 1024 | 11.8 | 8.6 | Angola | 353 | 13.2 | 15.1 | France, Guadeloupe | 74 | 44.4 | 30.5 |
| Germany | 8843 | 23.6 | 16.8 | Serbia | 1026 | 30.8 | 24.9 | Latvia | 404 | 51.5 | 41.1 | Kuwait | 57 | 6.8 | 10.9 |
| Italy | 8600 | 31.1 | 19.8 | Bulgaria | 995 | 34.3 | 26.9 | Cameroon | 334 | 13.7 | 15.2 | Trinidad and Tobago | 67 | 16.3 | 15 |
| Korea, Republic of | 7045 | 34.6 | 26.9 | Haiti | 888 | 53.1 | 51.7 | Denmark | 401 | 17.3 | 12.8 | Montenegro | 60 | 27.4 | 23.9 |
| Mexico | 5741 | 21.1 | 19.9 | Mali | 763 | 46.8 | 50 | Paraguay | 348 | 27.7 | 25.1 | Cabo Verde | 60 | 66.7 | 68.6 |
| Ukraine | 6207 | 38.2 | 34.2 | Iraq | 700 | 15.9 | 16.8 | Panama | 347 | 36.1 | 31.9 | Eritrea | 46 | 11.5 | 11.1 |
| Bangladesh | 5236 | 18.6 | 19.3 | Czechia | 903 | 21.7 | 16.3 | Albania | 358 | 36 | 31.3 | Luxembourg | 47 | 21.8 | 16 |
| Colombia | 5677 | 46.4 | 42.4 | Tanzania, United Republic of | 844 | 15.5 | 17.6 | Tunisia | 293 | 9.9 | 9.8 | Malta | 49 | 27.6 | 19 |
| Myanmar | 5448 | 49.5 | 53.2 | Cuba | 899 | 20.6 | 17.8 | Benin | 344 | 27.2 | 29.4 | Guinea-Bissau | 34 | 18.7 | 18.9 |
| Pakistan | 4514 | 15 | 15.5 | Yemen | 739 | 26.3 | 29.2 | Papua New Guinea | 330 | 28.3 | 34.8 | Qatar | 37 | 10.4 | 21.9 |
| Poland | 5247 | 36.5 | 28.9 | Austria | 810 | 21.8 | 15.2 | Jordan | 288 | 20.9 | 22.2 | Bahrain | 32 | 13.9 | 19.8 |
| Spain | 5060 | 26.2 | 18.4 | Kyrgyzstan | 691 | 62.3 | 68.7 | Uruguay | 322 | 29.4 | 22.8 | Namibia | 24 | 8.2 | 8.3 |
| France | 4718 | 18 | 12.6 | Belgium | 754 | 16.5 | 11.4 | Niger | 248 | 12.1 | 13 | Brunei Darussalam | 29 | 31.6 | 39.7 |
| Peru | 4513 | 60.7 | 52.5 | Costa Rica | 719 | 52.5 | 46.2 | Somalia | 247 | 16.9 | 17.7 | Gabon | 20 | 7.9 | 7.9 |
| United Kingdom | 4183 | 16.3 | 10.8 | Bolivia, Plurinational State of | 656 | 31.3 | 27.3 | Ireland | 317 | 20.2 | 15.3 | The Republic of the Gambia | 23 | 10.9 | 11.8 |
| Korea, Democratic Republic of | 3921 | 52.2 | 53.1 | Croatia | 688 | 39.5 | 29.1 | Slovenia | 308 | 35.1 | 24.8 | Timor-Leste | 26 | 14.8 | 15.2 |
| Chile | 3161 | 55.7 | 46.2 | Slovakia | 655 | 33 | 26.9 | Norway | 283 | 14.3 | 10.4 | Suriname | 22 | 17.1 | 16.7 |
| Romania | 3080 | 40.7 | 33.5 | Dominican Republic | 596 | 26.9 | 25.1 | Estonia | 275 | 52.8 | 40.4 | Fiji | 21 | 11.8 | 13.2 |
| Thailand | 2860 | 11.9 | 10.8 | Ghana | 566 | 15 | 16.2 | North Macedonia | 263 | 36.9 | 32.9 | France, New Caledonia | 23 | 30.5 | 28.2 |
| Argentina | 2947 | 25.7 | 21.6 | Madagascar | 491 | 16.8 | 18.1 | New Zealand | 243 | 14.3 | 11.1 | Botswana | 19 | 6.1 | 6.2 |
| Indonesia | 2552 | 4.5 | 4.9 | Singapore | 624 | 28.7 | 27.5 | Malawi | 207 | 12.7 | 13.8 | Lesotho | 20 | 6.6 | 6.5 |
| Philippines | 2612 | 13.9 | 14.6 | Mongolia | 522 | 94.4 | 107.9 | Zambia | 173 | 12.4 | 14.3 | Djibouti | 16 | 10.6 | 10.4 |
| Egypt | 2183 | 13.4 | 13.8 | Switzerland | 595 | 17 | 12.6 | Guinea | 189 | 15.3 | 15.5 | Guyana | 16 | 9.7 | 9.1 |
| Kazakhstan | 2184 | 50.4 | 50.8 | Sri Lanka | 554 | 9.1 | 8.7 | South Sudan | 201 | 17.1 | 17.9 | Barbados | 21 | 19.7 | 14.8 |
| Portugal | 2212 | 49.4 | 34.4 | Lithuania | 591 | 50.6 | 39.8 | Burundi | 197 | 20.2 | 21.5 | French Polynesia | 20 | 27.1 | 27.4 |
| Ethiopia | 1404 | 11.9 | 12 | Bosnia and Herzegovina | 570 | 42.9 | 36.6 | Burkina Faso | 220 | 12.2 | 13.3 | Bahamas | 19 | 19.5 | 20.7 |
| Nigeria | 1879 | 9.4 | 11 | Sudan | 417 | 8.3 | 8.6 | Lebanon | 164 | 11 | 11 | Saint Lucia | 19 | 37.5 | 31.4 |
| Uzbekistan | 1909 | 32.7 | 35.6 | Georgia | 537 | 38.9 | 33.7 | Chad | 146 | 11.2 | 12 | Equatorial Guinea | 12 | 10.4 | 10.8 |
| Morocco | 1687 | 20.8 | 20.7 | Lao People's Democratic Republic | 436 | 42.7 | 45.6 | Togo | 145 | 17.3 | 18.8 | Samoa | 12 | 37 | 39.8 |
| Ecuador | 1802 | 51 | 44.1 | Zimbabwe | 519 | 37.4 | 39.5 | Jamaica | 179 | 25.1 | 22.8 | Sao Tome and Principe | 9 | 38 | 42.7 |
| Belarus | 1848 | 53.1 | 47.4 | Sweden | 536 | 13.7 | 9.5 | Sierra Leone | 131 | 16.6 | 17.3 | Iceland | 15 | 12.9 | 9.4 |
| Canada | 1880 | 12.9 | 9.7 | El Salvador | 502 | 37.2 | 29.7 | Oman | 152 | 28.5 | 31.8 | French Guiana | 14 | 26.3 | 28.6 |
| Algeria | 1601 | 19.7 | 19.3 | Republic of Moldova | 508 | 39.6 | 37.6 | Libya | 137 | 12.5 | 13.9 | Belize | 14 | 22.5 | 21.5 |
| Afghanistan | 1636 | 47 | 50.9 | Senegal | 425 | 25.9 | 27.2 | Puerto Rico | 155 | 12.8 | 9.1 | Guam | 11 | 24 | 20 |
| Venezuela, Bolivarian Republic of | 1651 | 25.6 | 24.6 | Israel | 479 | 21.6 | 16.2 | Mauritania | 103 | 20.4 | 22.3 | Eswatini | 8 | 6.8 | 6 |
| Congo, Democratic Republic of | 1484 | 17.4 | 18.6 | Saudi Arabia | 405 | 7.7 | 9.5 | Liberia | 92 | 16.8 | 17.7 | Solomon Islands | 4 | 5 | 5.3 |
| Kenya | 1314 | 26.1 | 31.1 | Rwanda | 410 | 29.5 | 30.8 | United Arab Emirates | 99 | 8.9 | 18.6 | Vanuatu | 1 | 2.6 | 3 |
| South Africa | 1247 | 12.5 | 13 | Honduras | 449 | 31.8 | 31.1 | Gaza Strip and West Bank | 95 | 17.3 | 18.8 | Comoros | 5 | 5.2 | 5.8 |

**Note.**

The mortality rate was the number of gastric cancer deaths per 100,000 individuals.

**Abbreviations.**

CMR, crude mortality rate; ASM, age-standardized mortality rate.
